# Validation of Olfactory Network Based on Brain Structural Connectivity and Its Association With Olfactory Test Scores

**DOI:** 10.3389/fnsys.2021.638053

**Published:** 2021-04-13

**Authors:** Alexander Wieck Fjaeldstad, Franz Stiller-Stut, Carsten Gleesborg, Morten L. Kringelbach, Thomas Hummel, Henrique M. Fernandes

**Affiliations:** ^1^Flavour Institute, Department of Clinical Medicine, Aarhus University, Aarhus, Denmark; ^2^Flavour Clinic, Department of Otorhinolaryngology, Holstebro Regional Hospital, Holstebro, Denmark; ^3^Center for Eudaimonia and Human Flourishing, University of Oxford, Oxford, United Kingdom; ^4^Interdisciplinary Center for Smell and Taste, Department of Otorhinolaryngology, TU Dresden, Dresden, Germany; ^5^Center of Functionally Integrative Neuroscience, Aarhus University, Aarhus, Denmark; ^6^Center of Music in the Brain, Aarhus University, Aarhus, Denmark

**Keywords:** structural connectivity, olfaction, olfactory significance, brain dynamics, olfactory connectome

## Abstract

Olfactory perception is a complicated process involving multiple cortical and subcortical regions, of which the underlying brain dynamics are still not adequately mapped. Even in the definition of the olfactory primary cortex, there is a large degree of variation in parcellation templates used for investigating olfaction in neuroimaging studies. This complicates comparison between human olfactory neuroimaging studies. The present study aims to validate an olfactory parcellation template derived from both functional and anatomical data that applies structural connectivity (SC) to ensure robust connectivity to key secondary olfactory regions. Furthermore, exploratory analyses investigate if different olfactory parameters are associated with differences in the strength of connectivity of this structural olfactory fingerprint. By combining diffusion data with an anatomical atlas and advanced probabilistic tractography, we found that the olfactory parcellation had a robust SC network to key secondary olfactory regions. Furthermore, the study indicates that higher ratings of olfactory significance were associated with increased intra- and inter-hemispheric SC of the primary olfactory cortex. Taken together, these results suggest that the patterns of SC between the primary olfactory cortex and key secondary olfactory regions has potential to be used for investigating the nature of olfactory significance, hence strengthening the theory that individual differences in olfactory behaviour are encoded in the structural network fingerprint of the olfactory cortex.

## Introduction

Central processing of olfactory stimuli is a complicated endeavour involving several cortical and subcortical regions. Although elements of how this extraordinary sense functions have been discovered in recent years, much more remains to be learned before we fully understand how olfactory perception functions in humans.

A challenge in understanding the links between olfactory behaviour and the measurable cortical responses is that individuals suffering from olfactory loss reveal quite heterogeneous behavioural responses to their impairment: while some report a manifestly reduced quality of life, others are untouched by the impairment (Miwa et al., 2001; Croy and Nordin, 2014). As such, the relationship between measurable olfactory dysfunction and the degree of perceived impairment and consequence seem to be anything but linear. Even in individuals with a normal sense of smell (normosmics), there is a large degree of variation in olfactory threshold (Cometto-Muñiz et al., 2008). Some odours are even undetectable for otherwise normosmic individuals, e.g., cilantro (Eriksson et al., 2012). Furthermore, the ability of normosmics to rate their own olfactory sensitivity is poor (Landis et al., 2003), demonstrating a large degree of variation in both olfactory abilities, significance of olfaction, and awareness of olfactory stimuli (Lötsch and Hummel, 2019; Oleszkiewicz and Hummel, 2019).

Different neuroimaging methods are utilised to add unique pieces of the puzzle to the temporal and organisational dynamics of how olfactory information is processed in the brain. However, in order to adequately combine information from functional and structural neuroimaging studies, a key prerequisite is that the studies are comparable. Several templates for defining the parcellation of the primary olfactory region exist, often with insufficient or little overlap between them, rendering a direct comparison impossible.

In 2017, a parcellation of the primary olfactory cortex (POC) was published, combining anatomical information with a meta-analysis of functional regions of olfactory activation (Croy et al., 2010). The structural connectivity (SC) of this novel primary olfactory cortical parcellation to key secondary cortical areas of olfaction was analysed to ensure the parcellation contained essential parts of the olfactory cortex. Although the parcellation has been applied in numerus later olfactory neuroimaging studies, the reproducibility of the structural olfactory fingerprint has never been tested. As such, a validation study of the olfactory cortical parcellation is warranted in order to ensure feasibility of this parcellation for future studies in the field.

If a stabile olfactory fingerprint of SC exist, differences in the connectivity pattern may help understand how olfactory function differs between both in patients with different phenotypes of olfactory loss and the wide spread of behavioural olfactory differences within a neurotypical population, such as olfactory test scores or individual significance of olfaction.

This aspect of individual olfactory significance has been quantified in the questionnaire “Individual significance of olfaction,” where it has been shown to have a large degree of variation (Fjaeldstad et al., 2017). Here, elements of olfactory significance are rated in 20 questions, covering aspects of olfactory inputs, ranging from olfactory association, to application of olfaction in everyday life, to consequences and decision-making. It is unknown, if the behavioural aspects of individual olfactory significance are related with changes in the underlying brain connectivity of primary and secondary olfactory brain regions. If so, this could give rise to significantly different brain dynamics and propose an alternative explanation for discrepancies in relationship between olfactory function and the impact olfaction has on our behaviour.

By reapplying this recently published method of analysing the SC of the key olfactory brain regions (Croy et al., 2010), we aimed to validate the SC patterns of the structural olfactory fingerprint parcellation. Furthermore, in an exploratory analysis, we aim to investigate the differences in SC related to increased individual significance of olfaction and olfactory test scores in a neurotypical normosmic population. Specifically, we aim to explore if increased olfactory association, application, and consequence were associated with meaningful differences in connectivity patterns between the primary and secondary olfactory cortices.

## Results

A SC matrix was created for both groups by combining diffusion data with an anatomical atlas and advanced probabilistic tractography. This allowed the robust estimation of a connectivity fingerprints that constitute the structural scaffolding subserving olfactory processing. The brain areas forming the final group fingerprint of SC was defined based on the set of target regions that showed a connection to the primary olfactory cortical (OC) seeds in more than 50% of the subjects [i.e., a consistent olfactory cortical network (OCN) target connection]. Our results derived from a cohort of 30 subjects, validate the previously reported olfactory SC fingerprint (secondary olfactory anatomical targets with direct connection to the OC), derived from a lower sample size, thus confirming the specificity and robustness of the anatomical signatures of SC linking the primary and secondary olfactory processing cores.

To explore if a group in high and low olfactory significance (LOS) scores resulted in differences in SC, participants were divided into two significantly different groups (*p* < 0.0001) based on their olfactory significance scores, see Table 1. Additional divisions into low and high groups of olfactory test scores and subjective olfactory function were also analysed, see Supplementary Table 2. The olfactory test scores (Sniffin’ Sticks threshold, discrimination, identification, and combined) and subjective olfactory function was not associated with increased SC between the primary and secondary olfactory cortices. However, total olfactory significance score was associated with increased SC strength to parahippocampus bilaterally and left amygdala.

**TABLE 1:** Demographics for the total cohort, the olfactory significance groups and the differences between groups.

	**Total cohort (*n* = 30)**	**Low olfactory significance (LOS) (*n* = 15)**	**High olfactory significance (HOS) (*n* = 15)**	**Confidence interval**	***p*-value**
Sex (M/F)	12/18	10/5	2/13	–	0.0078
Age (mean)	38.1	40.5	35.8	[33.4;42.9]	0.3228
TDI score	35.3	33.3	37.2	[32.7;37.8]	0.1199
Individual significance of olfaction score	57.1	50.2	64.1	[54.2;60.1]	<0.0001

*Fisher’s Exact test was used to assess differences in categoric variables and two-tailed *t*-test was used for continuous variables.*

### Validation of the Olfactory Fingerprint Parcellation

**FIGURE 1:**
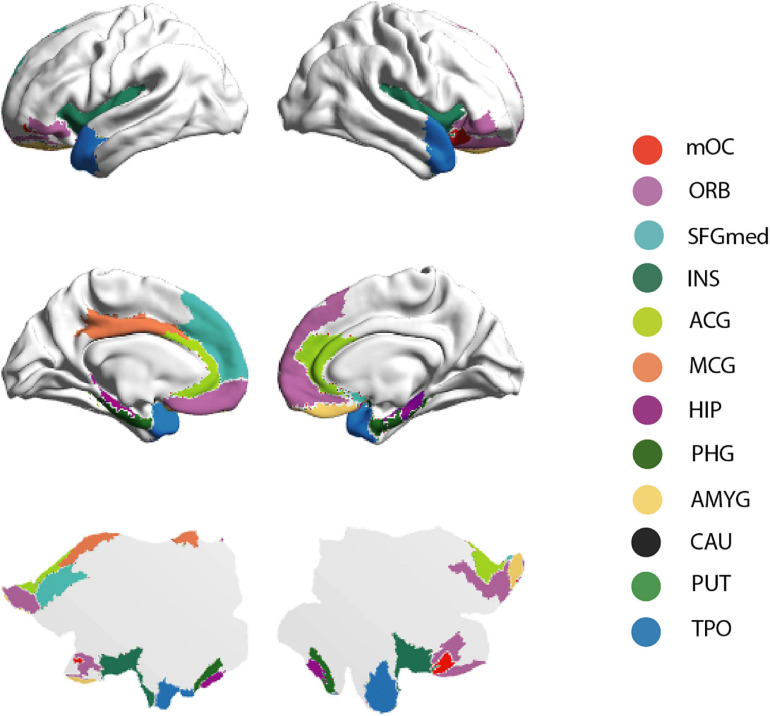
Brain rendering bistability of the OC fingerprint. Regions identified as part of the group OC fingerprint are projected into the cortical surface of the human brain—HCP atlas approach, here represented by 3-dimensional (“midtickness”: top and middle row) and 2-dimensional (“flatmap”: bottom row) mesh versions. Please note the inflated surface of the human brain here represented (Glasser360) does not include subcortical anatomy (Glasser et al., 2016).

The connectivity pattern of the OC fingerprint contained connections to all secondary olfactory areas of the previously published parcellation, see Figure 1. Furthermore, in this larger cohort, we found additional connections to the medial section of the superior frontal gyrus, bilaterally, and the left middle cingulate gyrus.

### Intra- and Interhemispheric Connectivity in Relation to Olfactory Significance

**FIGURE 2:**
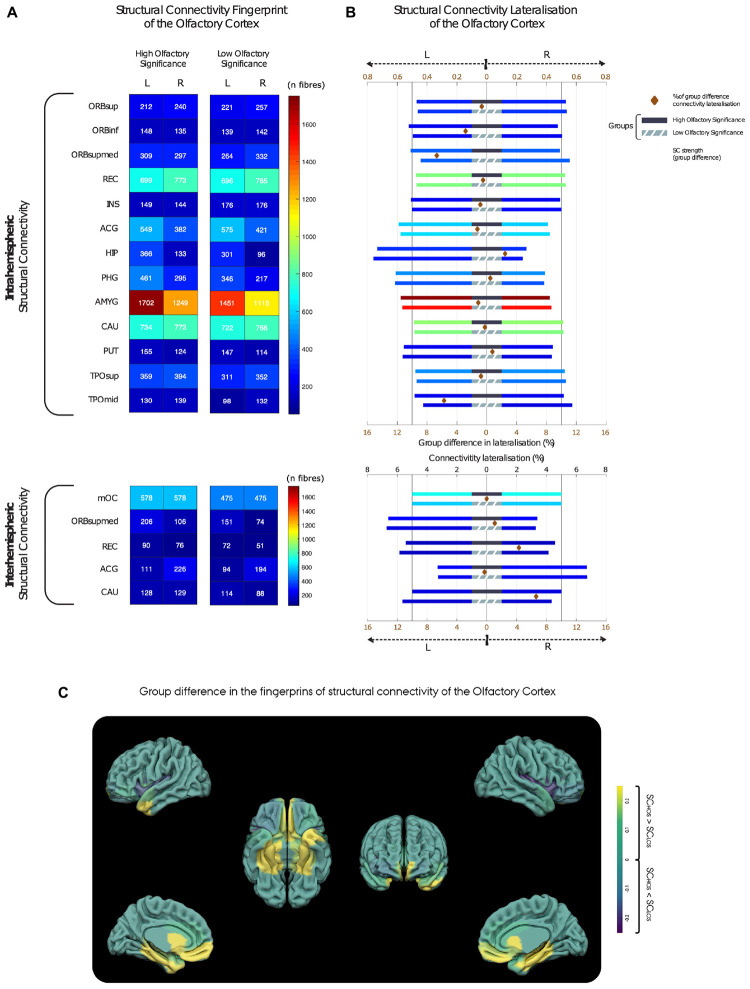
Inter- and intrahemispheric patterns of structural connectivity in the high and low olfactory significance group. **(A)** Structural connectivity strength between the olfactory cortex (OC) and the secondary olfactory regions. **(B)** Lateralisation of total structural connectivity for the high (top bars with black middle band) and low olfactory significance groups (bottom bars with grey middle band). Each bar has length of 1 with a vertical bar at 0.5 emphasising symmetric distribution of connectivity. To highlight differences in group lateralisation, a brown diamond between group bars represents the difference between HOS and LOS structural connectivity lateralisation [see bottom axis (brown) for percentage scale]. Notice that for the four regions had higher than 4% connectivity lateralisation difference, all showing a pattern of more symmetric laterality distribution of connectivity in the HOS group. These major group differences were found in frontal medial orbital, medial temporal pole, rectus, and caudate regions. **(C)** Anatomical localisation of the OC (left) and the structural connectivity differences between groups (right). Yellow is representing higher connectivity in the HOS group, and blue lower connectivity as compared to the LOS group. HOS, High olfactory significance group; LOS, Low olfactory significance group. For brain region abbreviations, see Supplementary Table 3.

The group average OC fingerprints of SC show that, compared with the LOS group, the high olfactory significance (HOS) group reveals overall trends for increased intra-hemispheric connectivity, (see Figure 2A and Supplementary Table 1). Additionally, our results suggest the existence of a subtle intra-hemispheric structural connection between the left OC and the thalamus, which is specific to the HOS group.

With regards to lateralisation, our group-average results indicate that the intra-hemispheric structural connections between the OC and hippocampus were found to have the highest degree of lateralisation, as 73% of the total SC fibres are situated in the left hemisphere. Furthermore, a trend for lateralisation (left-hemisphere) was found in intrahemispheric connections of the OC to several regions, for both groups, which may be indicative of hemispheric OC structural network asymmetries. These included connections to the parahippocampus, amygdala, putamen, and anterior cingulate gyrus (ACG; Figure 2B).

Interhemispherically, the ACG displayed a tendency for right lateralisation with 67% of crossing connections linking the right OC to the left ACG. Mean group differences in lateralisation were relatively small. Four regions revealed higher than 4% group difference in lateralisation, all showing a pattern towards increased symmetry in the distribution of hemispheric connectivity in the HOS group. These group differences were found in frontal medial orbital, medial temporal pole, rectus, and caudate regions.

### Structural Connectivity Correlation Analysis

We investigated how SC of the olfactory SC fingerprint correlates with olfactory threshold, discrimination, identification, subjective olfactory function, and olfactory significance (see Supplementary Table 2). Furthermore, we studied if the different aspects of olfactory significance (application, association, and consequence) were associated with specific neural signatures of SC involving primary and key secondary olfactory regions in the brain (see Table 2).

**TABLE 2:** Differences in the olfactory cortical network (OCN) connectivity for olfactory significance subscales.

**SC (sum)**	**Application**		**Association**		**Consequence**	
**Brain areas**	**rho**	**pval**	**rho**	**pval**	**rho**	**pval**
ORB	–0.296	0.112	–0.144	0.456	–0.096	0.601
SFGmed	–0.219	0.241	–0.260	0.157	–0.262	0.164
INS	–0.111	0.562	–0.228	0.224	–0.275	0.143
ACG	–0.273	0.148	0.064	0.739	–0.246	0.192
MCG	–0.228	0.227	–0.057	0.774	–0.137	0.443
HIP	0.092	0.650	**0.405**	**0**.**027**	**0.403**	**0**.**024**
PHG	**0.373**	**0.042**	0.352	0.063	**0.446**	**0.012**
AMYG	0.189	0.328	0.319	0.087	**0.458**	**0.012**
CAU	0.107	0.578	–0.081	0.676	0.159	0.406
PUT	0.059	0.757	–0.061	0.758	0.074	0.690
TPO	–0.053	0.784	0.110	0.533	0.123	0.528

*pval, *P* value; ORB, Orbitofrontal cortex (including subregions: ORBsup, ORBinf, SFGmed, ORBsupmed, and REC); TPO, Temporal pole (including subregions: superior temporal gyrus, middle temporal gyrus). *Post hoc* permutation tests (based on Pearson’s linear correlation coefficient) were performed to address the problem of multiple comparisons. Bold text highlights significant correlations. For brain region abbreviations, see Supplementary Table 3.*

Our findings show that while there were no significant associations between olfactory test scores or subjective assessment of olfactory function and the SC of the OCN, the olfactory significance score correlates with an increased connectivity to the parahippocampus bilaterally (left: *r* = 0.39; *p* = 0.035; right: *r* = 0.47; *p* = 0.010) and the left amygdala (*r* = 0.36; *p* = 0.047) (see Supplementary Table 2).

### Subscales of Olfactory Significance

The “*Individual significance of olfaction*” questionnaire contains three subscales with six questions on olfactory association, olfactory application, and olfactory consequence. Here, we investigated the relationship between these olfactory subscales and the patterns of connectivity between the POC and secondary olfactory brain regions (bilaterally, i.e., sum of the left and right OC fingerprints of SC) (see Figure 1 and Table 2).

Our results suggest that the SC strength between the OC and a group of secondary olfactory regions is significantly and positively correlated with olfactory application (parahippocampus: *r* = 0.373; *p* = 0.042), association (hippocampus: *r* = 0.405; *p* = 0.027), and consequence (hippocampus: *r* = 0.403; *p* = 0.024; parahippocampus*: r* = 0.446; *p* = 0.012; amygdala: *r* = 0.458; *p* = 0.012). All *p*-values presented were adjusted using permutation tests based on Pearson’s linear correlation coefficient to control the family-wise error rate (FWER; Groppe et al., 2011; Groppe, 2021).

**FIGURE 3:**
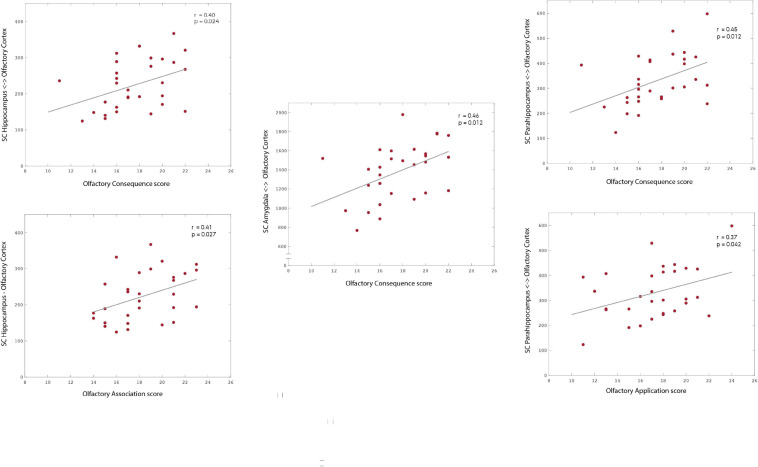
Association between subscales of olfactory significance—application, association, and consequence—and the structural connectivity to areas of the OC fingerprint. Our results suggest the existence of an association between olfactory significance scores and the structural connectivity strength to the mOC. Specifically, to the hippocampus (association: *r* = 0.405; *p* = 0.027; consequence: *r* = 0.403; *p* = 0.024), parahippocampus (application: *r* = 0.373; *p* = 0.042; consequence: *r* = 0.446; *p* = 0.012) and amygdala. Please notice the different ranges of structural connectivity on the *y*-axis. The *p*-values are adjusted using permutation tests based on Pearson’s linear correlation coefficient to correct for multiple comparisons.

Additionally, we investigated the potential association between each of these olfactory significance subscales and gender. Our results indicate that, across all three subscales, there is a slight tendency for women to score higher (mean ± standard deviation; application: 17.67 ± 3.33; association: 19.44 ± 2.81; consequence: 18.33 ± 2.52) than men (application: 16.42 ± 3.09; association: 16.75 ± 1.82; consequence: 16.42 ± 2.91). However, our results from (unpaired) *t*-tests indicate that, while there is no statistical gender difference in application (*p* = 0.065) and consequence (*p* = 0.065), association rating is significantly higher in women compared to men (*p* = 0.007) (see Figure 3). However, we recognise that the uneven gender distribution of this cohort (male/female: 12/18) may be importantly driving the statistical differences in gender here presented.

## Discussion

Our study validates the previously reported olfactory SC fingerprint derived from a smaller sample, thus supporting the existence of a specific and robust structural network linking the primary and secondary olfactory cores.

In our exploratory analysis, our results indicate that olfactory significance may be associated with differences in the configuration and strength of SC in the OCN, although gender may be an influencing factor. Additionally, higher levels of olfactory significance were linked to the existence of a subtle structural connection between the thalamus and the OC. We found a positive association between increased degree of individual olfactory significance and increased SC. Specifically, different subscales of olfactory significance were associated to the SC between the POC and the hippocampus (consequence), parahippocampus (application and consequence), and amygdala (consequence).

### Validation of the Olfactory Fingerprint Parcellation

In our sample of 30 participants, we validated the existence of connections from the olfactory parcellation to all secondary olfactory regions identified in the original publication. Given the robustness of the SC pattern, the mOCN template has great potential for future use in olfactory research, as it is applicable in both structural and functional neuroimaging studies.

In this larger cohort, we found additional connections to two brain regions, the medial section of the superior frontal gyrus, bilaterally, and the left middle cingulate gyrus. While the literature on these regions in relation to olfaction is limited, they may be relevant for further investigation; The superior frontal gyrus is a supplementary motor area is underactive in Parkinson’s disease, where olfactory loss is a dominant trait. The middle cingulate gyrus is a limbic region described to be activated from the midline, mediodorsal and intralaminar thalamic nuclei which evoke fear and mediates nocifensive behaviours (Vogt, 2016), a behavioural trait often affected by olfactory input. Further research is required to understand the importance and effect of these connections.

### Lateralisation of Structural Olfactory Brain Connectivity

For all participants, the intra-hemispheric connectivity between the primary and secondary olfactory regions were stronger on the left hemisphere. Furthermore, we found the existence of interhemispheric connectivity between the POC and several contralateral regions, including the POC and several key secondary olfactory regions. The connectivity strength was more balanced between hemispheres in the HOS group, who showed a higher interhemispheric connectivity between primary and secondary olfactory regions, see Figures 2A,B. This is in line with previous functional studies, where initial ipsilateral activation of the POC after a short delay is followed by activation in the contralateral hemisphere, which may indicate connectivity between the two hemispheres in the anterior commissure (Lascano et al., 2010; Stadlbauer et al., 2016).

Lateralisation of olfaction is known to exist in animal models (Teitelbaum, 1971; Zatorre et al., 1992). More recent animal studies have shown increased behavioural effects by unilateral deprivation of the left olfactory system as compared with the right olfactory system (Kishimoto et al., 2013).

Previous studies in humans also reported olfactory lateralisation in olfactory processing. Initial studies in humans focused on the investigation of the effects of unilateral brain damage. Left-hemisphere damage was associated with increased difficulties in olfactory identification tasks (Bellas et al., 1989), which may be related to language processing. Hudry et al. (2014) found that patients with left temporal pole epilepsies rated odours as less pleasant and had increased difficulties with olfactory identification, compared to patients suffering from right temporal pole epilepsies. Another study on patients with unilateral anterior medial temporal lobe resections, left-sided resected patients were found to have a decreased emotional saliency in response to odours (Juran et al., 2016). However, older studies reported indications of right-sided olfactory dominance, as patients with right temporal lobe with right orbitofrontal lesions performed poorly in odour recognition tests as compared with patients with equivalent left-sided lesions (Rausch et al., 1977; Jones-Gotman and Zatorre, 1993). On the other hand, a more recent study found bilateral impairment after unilateral anterior pole lobectomy (Haehner et al., 2012). As indicated by these contradictory findings of olfactory impairment after unilateral brain lesions, olfactory processing seems to differ between individuals and may depend strongly on both intra- and inter-hemispheric processing. It could be that these connections become more important for more complex tasks, like odour discrimination which involved application of odour memory (Kupfermann, 1991; Hummel et al., 1998).

In recent years, neuroimaging studies on healthy individuals have contributed to the investigations of olfactory lateralisation. A study on olfactory electrophysiological responses, led by Cohen et al. (2015), demonstrated a robust interhemispheric asymmetry in the activity of the anterior piriform cortex. This asymmetry emerged during specific stages of odour discrimination learning, with a transient bias towards the left hemisphere (Cohen et al., 2015). The nature of the olfactory stimuli and study paradigm seems to have profound effects on the pattern of cortical activation and lateralisation. This is evident from the comprehensive review by Saive et al. (2014) on episodic odour memory. Here, different patterns of left and right-sided activation were found in numerous studies on aspects such as odour familiarity, recognition, episodic, and autobiographical memory.

The patterns of SC have a high degree of temporal stability (Cheng et al., 2012; Park and Friston, 2013), while cortical activation and olfactory sensitivity during functional olfactory neuroimaging can be affected by confounders such as hunger (Hanci and Altun, 2016), attention (Zelano et al., 2004), noise (Fjaeldstad et al., 2019), sleepiness (Ghielmini et al., 2013), order of odour presentation (Walliczek-Dworschak et al., 2016), endogenous variation (Hoffmann, 2019), and order of odour exposure (Cecchetto et al., 2019). Therefore, compared to earlier functional neuroimaging studies on olfactory lateralisation, the current methodology of SC offers a more stable assessment of olfactory lateralisation and a structural framework for understanding potential functional effects.

### Relevance of Olfactory Significance

We are constantly bombarded with sensory stimuli. In complex neural networks, the significance of these inputs differs depending on context and prediction where only the relevant sensory inputs alert our attention (Fjaeldstad et al., 2016). From an evolutionary perspective, all senses have a role to play. Especially highly salient or unexpected stimuli can alter attention. These are key in determining the spatial location of the source, being either prey or predator (Knudsen, 2018). This can be initiated by auditory or visual clues (García-García et al., 2013; Papies, 2013), however, olfactory stimuli are among the most potent triggers of attention - both aversion and attraction (Li and Liberles, 2015).

Environmental attention is a computation applied to competing sensory information in order to selectively apply focus and avoid distractions from alternative sensory input (Amso and Scerif, 2015). However, attention is not a constant or unitary process, it is a dynamic balance that can be captured by salient or unexpected events (bottom-up, stimulus driven attention) as well as deployed under voluntary control (top-down, goal directed attention). The assessment of sensory input rests upon a dynamical balance of brain processing which seems to exhibit a large degree of individual variability. As such, an identical sensory stimulus can elicit responses with different hedonic valence, behavioural response, and sensitivity of perception. As the neural pathway of olfactory input differs from other senses by initially circumventing the thalamus (Shepherd, 2005), the ratings of individual olfactory significance may offer insights to how odours in our environment are mirrored in our behaviour.

Prior experiences and context seem to play an important role in sensory perception. As suggested by Friston, the brain dynamics can be described as a predictive coding framework, where the sensory prior experience plays an important role in both interpretation and energy allocation when determining how to respond to a stimulus. With predictive coding, all incoming signals are compared with previous experience (“priors”). If the sensory stimulus differs from these priors, sensory event related potentials as equivalents of brain activity exhibit an alteration—a phenomenon known as mismatch negativity (Friston, 2005). This is seen in olfactory processing, where the brain responses of experts are more minute and localised as compared with novices (Plailly et al., 2012). However, perception cannot copiously be described using only differences between inputs and priors, as the relevance of the sensory input for our current goals. Parameters such as attention, value, reinforcement, and salience can be important modulators of responses to sensory stimuli (Friston, 2009). As such, responses to olfactory stimuli differ among individuals, where some are more prone to use their sense of smell in their everyday chores (application), their reflections when perceiving odours (association), and the impact odours inflict on behaviour (consequence) (Croy et al., 2010, 2012).

### Areas With Altered Structural Connectivity in Individuals With High Olfactory Significance

During olfactory stimulation, the initial activation in both hemispheres occurs in the POC, hippocampus, parahippocampus, amygdala, and orbitofrontal cortex (Stadlbauer et al., 2016). As such, these regions are highly important for secondary olfactory processing.

#### Olfaction and Memory

The information processing pathways of memory processing involves a series of connections including the trisynaptic circuit linking the parahippocampus and hippocampus in consolidating memory from patterns of cortical activation (Witter et al., 2000). The amygdala is reciprocally connected to a large extension of both the hippocampus and parahippocampus (McDonald and Mott, 2016). Projections from the basolateral amygdala to the hippocampus are key in modulating the consolidation of memories of emotionally arousing experiences (McGaugh, 2004), as well as for enhancing explicit memories of emotionally arousing events (Phelps, 2004). The connections between amygdala, hippocampus, and parahippocampus are also activated during retrieval of emotional memories (Dolcos et al., 2005). In comparison with other sensory modalities, odours are considered powerful triggers of emotional memories with a high degree of stability over time (Saive et al., 2014) and seem to be strengthened by the hedonic valence of an odour (Larsson et al., 2009).

#### Hippocampus

The hippocampus is a key structure for memory, where short-term memory is consolidated into long-term memory. In general, the ventral part of hippocampus is assumed to be mainly involved in stress, emotion, and affect, while the dorsal hippocampus performs cognitive function (Fanselow and Dong, 2010; Strange et al., 2014). Hippocampal activation occurs shortly after olfactory stimulation (Stadlbauer et al., 2016) and is assumed to play a key role in secondary olfactory processing related to olfactory learning and memory (Arshamian et al., 2013).

#### Parahippocampus

The parahippocampus is also closely linked to memory. However, olfactory memory processing seems to be affected by odour context, as odour familiarity can affect the neural processing. Familiarity is key for semantic association and episodic retrieval functions (Plailly et al., 2007) and has been strongly correlated specifically with parahippocampal activation (Savic and Berglund, 2004).

#### Amygdala

A part of the amygdala receives direct input from the olfactory bulb. As such, this sub-region is by definition a part of the POC (Carmichael et al., 1994) and thus included in the OC. All odours—independent of hedonic valence - seems capable of activating of amygdala (Gottfried et al., 2002). However, a difference in processing of odour input within amygdala has been strongly linked to hedonic valence of odours and emotional behaviour (Lehman et al., 1980; Small et al., 1997; Patin and Pause, 2015).

#### Thalamus

We found the existence of connectivity between the OC and the thalamus in individuals with an increased significance of olfaction. In general, the thalamus is regarded as having a key role in sensory attention (Halassa et al., 2014). However, as the only sensory modality, olfactory input bypasses the thalamic relay before reaching the primary sensory cortex (Shepherd, 2005). As such, the role of olfactory attention and olfactory consciousness is unique from a sensory perspective (Keller, 2011). Some modulation of attention seems to be processed directly in the anterior part of the POC, which is key for focussed sniffing for odours and anticipatory responses (Zelano et al., 2004).

Although the thalamus is bypassed in the direct pathway from the olfactory bulb to the POC, thalamic activity still seems to be of importance for conscious analysis of olfactory input (Plailly et al., 2008). The level of odour-related arousal can be an important factor for determining the level of thalamic activation (Sorokowska et al., 2016). While continuous olfactory stimulation is known to cause desensitisation, this decrease in olfactory responsiveness can be modulated by olfactory attention (Fallon et al., 2018). As such, our findings support that olfactory attention may be partly regulated by the thalamus, which falls within the framework of recent animal studies (Courtiol, 2019) and human functional olfactory studies (Zhou et al., 2019).

#### Subscales of Olfactory Significance

Our findings suggest that there are specific patterns of association between the SC of the POC to different key secondary olfactory areas, and each of the different functional components of olfactory significance. Olfactory application was positively correlated with increased connectivity to parahippocampus, olfactory association was positively correlated with increased connectivity to hippocampus, while olfactory consequence was positively correlated with increased connectivity to hippocampus, parahippocampus, and amygdala. As such, these patterns could reflect different underlying processing of olfactory stimuli and memory. Moreover, for individuals with a high olfactory consequence score, the increased connectivity to amygdala provides a neurostructural basis for the increased olfactory driven emotional behaviour. However, the high and LOS groups were not gender balanced and female participants has significantly higher olfactory association sub-scores of olfactory significance.

### Limitations

Given the design of the study, no firm conclusions can be drawn regarding the underlying cause of the significant changes in connectivity profiles found in individuals with higher olfactory significance. The current study did not investigate whether the participants with increased olfactory sensitivity also were more sensitive to other sensory modalities. As such, our study contributes with a scaffold of structural olfactory connections linking both individual olfactory significance with structural differences, but also linking previous functional olfactory studies with intra- and interhemispheric connectivity patterns regardless of olfactory significance grouping. Participants in the HOS and LOS group were not sex matched. The higher proportion of female participants in the HOS group is not surprising, as women have been found to outperform men in olfactory abilities (Sorokowski et al., 2019). The HOS had both higher intra- and interhemispheric SC, which has not previously been described as a gender-specific pattern of connectivity, as previous studies have suggested that males in general have higher structural intrahemispheric connectivity and females have greater interhemispheric connectivity (Ingalhalikar et al., 2014; Xin et al., 2019). There are several possible explanations for the identified differences in SC: changes in structural olfactory connectivity are associated with olfactory significance, gender, or a combination of these two parameters. Regardless of which explanation is driving the difference, this is a novel and very relevant point that is highly relevant to explore in future larger studies. Some of these potential associations can be explored in the open access databases, such as the Human Connectome Database, where participants have been screened for olfactory function with a nine-item olfactory identification test. However, more detailed information on olfactory function may be required to untangle the underlying dynamics of changes in olfactory connectivity. While more detailed information on olfactory sensitivity using the TDI score is recommended, other aspects of olfactory sensitivity are also recommended to be considered, e.g., perception of odorant mixtures (Lovitz et al., 2012; Oleszkiewicz et al., 2016), olfactory perceptual space (Fournel et al., 2016), and sensitivity to other key senses involved in the multisensory processing of food (gustatory and intranasal trigeminal).

The current methodology was applied to investigate differences in SC, as this has an advantage in terms of temporal stability. However, to further understand the functional dynamics related to changes in individual significance, it would be relevant to study the functional olfactory pathways in relation to olfactory significance (Zhou et al., 2019).

In order to investigate whether the association between connectivity patterns and olfactory significance is innate or learned, longitudinal studies are necessary.

## Conclusion

In summary, the structural network of olfactory connectivity was reproduced from earlier findings (Fjaeldstad et al., 2017), confirming the validity of the primary olfactory parcellation. The validates that the merged and updated olfactory parcellation from previous anatomical and functional olfactory parcellations does prove robust and reproducible. As such, the olfactory parcellation enables a direct comparison between structural and functional neuroimaging studies using this parcellation.

Furthermore, differences in connectivity strength revealed potential associations between SC of the OCN and individual significance of olfaction. In our exploratory analysis, participants with higher ratings of olfactory significance had increased intra- and inter-hemispheric SC. Furthermore, the patterns of SC differed between the POC and key secondary olfactory regions, depending on the nature of olfactory significance. While the differences in SC are not conclusive and the analysis was exploratory and not gender matched, the SC patterns may prove a useful tool for investigating the relationship between cortical networks and behaviour.

These findings can drive hypotheses for future studies. Further research is needed to investigate if the changes in lateralisation are attributed to learned or innate properties and to ensure that elements of the current findings are not driven by gender effects.

## Materials and Methods

We estimated the fingerprint of SC involving primary and secondary areas in olfactory processing by applying probabilistic tractography on diffusion tensor imaging (DTI) data. By combining the underlying measures of diffusivity, with a whole-brain anatomical atlas, estimation for crossing fibres and a tractography algorithm, a full map of whole-brain structural neural networks can be compiled. This algorithm has previously been successfully applied to merge findings from functional and structural olfactory studies to identify an OCN and construct a new parcellation of the POC by merging structural and functional templates [merged olfactory cortex, referred in this manuscript as the olfactory cortex (OC)] (Fjaeldstad et al., 2017). This merged OC includes the olfactory regions from the Automated Anatomical Labelling (AAL) parcellation (piriform and entorhinal cortex) and the adjacent olfactory part of the amygdala (Figure 2C).

### Participants and Ethics

All 30 study participants were healthy, right-handed, and between 24 and 62 years of age. Participants with a subjective normal or increased sense of smell were recruited. All participants filled out the “Individual significance of olfaction” questionnaire (Croy et al., 2010). Depending on their scores in this questionnaire, participants were divided into groups of high olfactory significance and low olfactory significance, HOS and LOS, respectively. Their subjective assessment of olfactory function was registered by having participants fill out their assessment of subjective olfactory function on a scale from 1 to 9 [9 (extremely well), 5 (normal), 1 (no perception)] and grouped into a high subjective smell group (score 6–9) and low subjective smell group (1–5). Participants who rated their olfactory function as better than normal (6–9) were categorised as subjective hyperosmic.

All participants underwent assessment of nasal patency and olfactory function. Olfactory function was evaluated using the Sniffin’ Sticks (including odour threshold, discrimination, and identification tests), where all participants were found to be normosmic.

Prior to inclusion, all participants received oral and written information on the study and signed an informed consent form. The study was conducted in accordance with the Declaration of Helsinki for medical research and approved by the ethics committee at the TU Dresden (Protocol # EK262082010).

### Olfactory Significance Sub-Scores

The individual significance of olfaction questionnaire can be sub-divided into three categories: association, application, and consequence (Croy et al., 2010).

The association scale is based on six questions and reflects emotions, memories, and evaluations that are triggered by olfaction. This includes questions such as “Certain smells immediately activate numerous memories.” The application scale consists of six questions and reflects how much olfaction is used daily. The consequence scale is based on six questions and reflects the importance attributed to olfaction in daily decisions. It includes questions such as “If my partner has a nasty smell, I avoid kissing him/her.”

### Neuroimaging

All participants underwent the acquisition of whole-brain T1-weighted and diffusion-weighted images using a 3T Skyra MRI (Siemens, Erlangen, Germany) equipped with a 32-channel head-coil, at the Universitätsklinikum Carl Gustav Carus, Dresden, Germany.

The 3D T1-weighted image was obtained from a MP2RAGE sequence (a magnetisation-prepared rapid gradient echo derivative), consisting of the following parameters: field-of-view of 256 mm × 256 mm reconstructed matrix; 1 mm × 1 mm in-plane resolution; slice thickness of 1 mm; TR/TE = 5000/2.96 ms; flip-angle of 4°, in-plane acceleration (iPat) factor of 2.

A full spin-echo EPI multi-shell dMRI session included the acquisition of two phase encoding directions with opposite polarities (anterior to posterior; posterior to anterior), represented by a single gradient table with 210 directions across five different non-zero b-values, with additional 9 b = 0 volumes. The diffusion weighting consisted of 5 shells of b (700, 1,000, 1,200, 1,500, 2,500 s/mm^2^), interspersed with a total number of acquisitions of 15, 21, 30, 60, and 75, respectively. All 210 directions were non-linear and uniformly distributed.

The acquisition parameters were: TE/TR = 70.80/2650 ms, flip-angle of 90°, resolution of 2 mm × 2 mm × 2 mm, with 78 slices, multiband factor of 3 and iPat factor of 2.

### Olfactory Fingerprinting Analysis

The measurement of SC is based on previously published methods of olfactory fingerprinting, see Fjaeldstad et al. (2017) for more detailed description. This method uses a probabilistic tractography algorithm which generates a connectivity distribution from all seed voxels in the AAL brain parcellation template (Tzourio-Mazoyer et al., 2002). The method is a based on an algorithm previously designed for identifying the whole-brain fingerprints of SC from deep brain stimulation electrodes in patients with chronic pain (Fernandes et al., 2015). The algorithm applied consists of three steps: compensating for orbital and sinus related distortions, fitting an anatomical atlas (AAL template in MNI space; 90 brain regions with the corrected olfactory cortex parcellation, OC) to each participant’s brain, estimating the SC between brain regions.

#### Compensating for Image Distortions

The FDT toolbox in FSL (version 5.0, http://www.fmrib.ox.ac.uk/fsl/, FMRIB, Oxford) was used to carry out the multi-stage processing pipeline of the diffusion MRI data. A first pre-processing stage involved the correction for head motion and image distortions induced by gradient eddy currents, predominantly affecting the orbitofrontal cortex and inferior temporal lobe. For this FSL tools EDDY and TOPUP combined the two diffusion datasets acquired in opposite phase-encoding directions, to produce a bias-field correction map subsequently applied to these datasets to optimise the signal in regions of signal dropout or field-induced distortions (Andersson et al., 2003).

#### Fitting an Anatomical Atlas to Each Individual Brain

Based on a modified version of the AAL brain atlas to include the POC (referred to in this manuscript as OC), the brain was parcellated into 90 subcortical and cortical regions (Tzourio-Mazoyer et al., 2002). We applied the FLIRT tool (FMRIB, Oxford, United Kingdom) (Jenkinson et al., 2002) for linear co-registration (12-parameter affine model) of the ICBM152 in MNI space into the participant’s T1 structural image using geometric registration and nearest-neighbour interpolation (Collins et al., 1994). The resulting transformation matrix that was concatenated with the T1 to DTI native space transformation matrix to allow for a direct spatial normalisation of the AAL template in MNI space to each subject’s diffusion native space.

#### Estimating Structural Connectivity

Subsequent modelling for crossing fibres within each voxel of the brain was performed using a Markov Chain Monte Carlo sampling algorithm. This allowed the estimation of the distributions on several diffusion parameters as well as the local probability distribution of fibre direction at each voxel of the brain, as described by Behrens et al. (2003) For this step, we used an automatic voxel-level estimation of two-fibre directions to increase the tracking sensitivity of non-dominant fibres in the brain (Behrens et al., 2007).

Probabilistic tractography at the voxel-level was used to estimate the probability of connectivity. A sampling number of streamline fibres per voxel was set at 5000. Brain boundaries were described based on a binary mask of each subject’s native brain space. Connectivity between a seed voxel *i* and a target voxel *j* was defined as the proportion of streamlines that leaving voxel *i* reached voxel *j* (Behrens et al., 2007). This was further extended from voxel to region level, in which 5,000 fibres were sampled for each voxel comprised in a brain region. The connectivity probability *P*_*ij*_ from *i* to *j* is then calculated as the number of sampled fibres in region *i* connecting the two regions, divided by 5,000 × *n*, where *n* is the number of voxels in region *i*. The connectivity probability to each of the remaining 89 regions was estimated for each area of the brain (defined by the AAL template). Regional connectivity was computed and further normalised by each area’s volume (number of voxels). Given the undirected nature of DTI measurements, and the high correlation between the estimated probability of connectivity from *i* to *j* and *j* to *i*, across all brain areas, we defined the undirectional connectivity probability *P*_*ij*_ between regions *i* and *j* by averaging these two values. This averaged connectivity value was used to characterise the SC strength between every OC seeds and the rest of the brain. For each participant, a 2 × 90 weighted matrix was constructed, representing the brain’s olfactory SC network for the right and left OC.

The brain areas forming the group olfactory fingerprint of SC was defined based on the set of target regions consistently connected to the OC region (i.e., with a target–OC connection appearing in more than 50% of the subjects). Subsequently, in order to validate the previously reported OC seed (Fjaeldstad et al., 2017) (using the same methodology), we verified if our cohort of 30 participants reproduced the same set of secondary targets—olfactory SC fingerprint—to which the OC was previously shown to be directly connected to.

#### Correlation Analysis—Structural Connectivity and Olfaction Scores

We computed the Pearson’s linear correlation coefficient to investigate how the SC strength of each of the brain areas comprising the previously reported olfactory SC fingerprint (Fjaeldstad et al., 2017) may predict olfactory threshold, discrimination, identification, subjective olfactory function, and olfactory significance. *Post hoc* permutation tests (based on Pearson’s linear correlation coefficient) were performed to address the problem of multiple comparisons (Groppe et al., 2011).

## Data Availability Statement

The raw data supporting the conclusions of this article will be made available by the authors, without undue reservation.

## Ethics Statement

The studies involving human participants were reviewed and approved by TU Dresden (Protocol # EK262082010). The patients/participants provided their written informed consent to participate in this study.

## Author Contributions

AF, TH and HF conceived the idea for the project. FS-S and TH performed the patient inclusion. AF wrote the initial draft for the manuscript. All authors contributed to the planning of the study and data acquisition and to a subsequent revision of the manuscript.

## Conflict of Interest

The authors declare that the research was conducted in the absence of any commercial or financial relationships that could be construed as a potential conflict of interest.
