# Supplementary material for: Validation of Olfactory Network Based on Brain Structural Connectivity and Its Association With Olfactory Test Scores
Source: Front Syst Neurosci. 2021 Apr 13;15:638053. doi: 10.3389/fnsys.2021.638053 (PMC8078209; doi:10.3389/fnsys.2021.638053)
Supplement: Supplementary file 1 [file Table_1.DOCX]

## **Supplementary tables**

| **Intra-hemispheric connectivity** | | | | | | | | |  | **Inter-hemispheric connectivity** | | | | | | | | |
| --- | --- | --- | --- | --- | --- | --- | --- | --- | --- | --- | --- | --- | --- | --- | --- | --- | --- | --- |
| **OC fibres, left** | | | |  | **OC fibres, right** | | | |  | **OC fibres, left** | | | |  | **OC fibres, right** | | | |
| **Targets** |  | **LOS** | **HOS** |  | **Targets** |  | **LOS** | **HOS** |  | **Targets** |  | **LOS** | **HOS** |  | **Targets** |  | **LOS** | **HOS** |
|  |  |  |  |  |  |  |  |  |  |  |  |  |  |  |  |  |  |  |
| Frontal_Sup_Orb |  | 221 | 212 |  | Frontal_Sup_Orb |  | 257 | 240 |  | Frontal_Sup_Orb |  | 16 | 19 |  | Frontal_Sup_Orb |  | 0 | 3 |
| Frontal_Inf_Orb |  | 139 | 148 |  | Frontal_Inf_Orb |  | 142 | 135 |  | Frontal_Inf_Orb |  | 2 | 0 |  | Frontal_Inf_Orb |  | 0 | 0 |
| Olfactory |  | 0 | 0 |  | Olfactory |  | 0 | 0 |  | Olfactory |  | 490 | 578 |  | Olfactory |  | 490 | 578 |
| Frontal_Sup_Med |  | 22 | 0 |  | Frontal_Sup_Med |  | 31 | 24 |  | Frontal_Sup_Med |  | 24 | 17 |  | Frontal_Sup_Med |  | 8 | 0 |
| Frontal_Mid_Orb |  | 264 | 309 |  | Frontal_Mid_Orb |  | 332 | 297 |  | Frontal_Mid_Orb |  | 169 | 206 |  | Frontal_Mid_Orb |  | 83 | 106 |
| Rectus |  | 696 | 699 |  | Rectus |  | 785 | 773 |  | Rectus |  | 80 | 90 |  | Rectus |  | 60 | 76 |
| Insula |  | 176 | 149 |  | Insula |  | 176 | 144 |  | Insula |  | 0 | 0 |  | Insula |  | 0 | 0 |
| Cingulum_Ant |  | 575 | 549 |  | Cingulum_Ant |  | 421 | 382 |  | Cingulum_Ant |  | 111 | 111 |  | Cingulum_Ant |  | 211 | 226 |
| Cingulum_Mid |  | 45 | 33 |  | Cingulum_Mid |  | 0 | 0 |  | Cingulum_Mid |  | 0 | 0 |  | Cingulum_Mid |  | 2 | 4 |
| Hippocampus |  | 301 | 366 |  | Hippocampus |  | 96 | 133 |  | Hippocampus |  | 0 | 3 |  | Hippocampus |  | 0 | 1 |
| ParaHippocampus |  | 346 | 461 |  | ParaHippocampus |  | 217 | 295 |  | ParaHippocampus |  | 0 | 0 |  | ParaHippocampus |  | 0 | 0 |
| Amygdala |  | 1451 | 1702 |  | Amygdala |  | 1115 | 1249 |  | Amygdala |  | 0 | 2 |  | Amygdala |  | 2 | 2 |
| Caudate |  | 722 | 734 |  | Caudate |  | 768 | 773 |  | Caudate |  | 115 | 128 |  | Caudate |  | 90 | 129 |
| Putamen |  | 147 | 155 |  | Putamen |  | 114 | 124 |  | Putamen |  | 0 | 2 |  | Putamen |  | 0 | 0 |
| Thalamus |  | 0 | 25 |  | Thalamus |  | 0 | 0 |  | Thalamus |  |  | 0 |  | Thalamus |  | 0 | 0 |
| Temporal_Pole_Sup |  | 311 | 359 |  | Temporal_Pole_Sup |  | 352 | 394 |  | Temporal_Pole_Sup |  | 0 | 0 |  | Temporal_Pole_Sup |  | 0 | 0 |
| Temporal_Pole_Mid |  | 98 | 130 |  | Temporal_Pole_Mid |  | 132 | 139 |  | Temporal_Pole_Mid |  | 0 | 0 |  | Temporal_Pole_Mid |  | 0 | 0 |
|  |  |  |  |  |  |  |  |  |  |  |  |  |  |  |  |  |  |  |
| **Mean** |  | 344 | 377 |  | **Mean** |  | 329 | 340 |  | **Mean** |  | 67 | 77 |  | **Mean** |  | 59 | 70 |
| **Std dev** |  | 370 | 421 |  | **Std dev** |  | 321 | 343 |  | **Std dev** |  | 129 | 152 |  | **Std dev** |  | 128 | 151 |
| **Mean_NZ** |  | 367 | 402 |  | **Mean_NZ** |  | 353 | 364 |  | **Mean_NZ** |  | 126 | 116 |  | **Mean_NZ** |  | 118 | 125 |
| **Sum** |  | 5511 | 6030 |  | **Sum** |  | 4937 | 5101 |  | **Sum** |  | 1006 | 1157 |  | **Sum** |  | 945 | 1127 |

**Supplementary Table 1.** Inter- and intra-hemispheric connectivity of the olfactory structural connectivity fingerprint and the thalamus in the low olfactory significance group (LOS) and high olfactory significance group (HOS).

LOS: Low olfactory significance group; HOS: High olfactory significance group; Std dev: Standard deviation; Mean_NZ: mean number of fibres excluding connections with zero fibres. For brain region abbreviations, see supplementary Table 3.

| **Targets** | **Olfactory significance** | | |  | **Subjective olfactory function** | | |  | **Combined olfactory TDI score** | | |  | **Olfactory threshold score** | | |  | **Olfactory discrimination score** | | |  | **Olfactory identification score** | | |
| --- | --- | --- | --- | --- | --- | --- | --- | --- | --- | --- | --- | --- | --- | --- | --- | --- | --- | --- | --- | --- | --- | --- | --- |
|  | **rho** |  | **pval** |  | **rho** |  | **pval** |  | **rho** |  | **pval** |  | **rho** |  | **pval** |  | **rho** |  | **pval** |  | **rho** |  | **pval** |
|  |  |  |  |  |  |  |  |  |  |  |  |  |  |  |  |  |  |  |  |  |  |  |  |
| **Frontal_Sup_Orb_L** | -0,222 |  | 0,238 |  | 0,232 |  | 0,212 |  | 0,053 |  | 0,802 |  | 0,004 |  | 0,984 |  | 0,010 |  | 0,938 |  | 0,151 |  | 0,420 |
| **Frontal_Sup_Orb_R** | -0,145 |  | 0,428 |  | 0,161 |  | 0,387 |  | -0,301 |  | 0,121 |  | -0,328 |  | 0,074 |  | -0,313 |  | 0,095 |  | -0,058 |  | 0,704 |
| **Frontal_Inf_Orb_L** | 0,062 |  | 0,758 |  | -0,128 |  | 0,502 |  | -0,153 |  | 0,431 |  | -0,146 |  | 0,441 |  | -0,165 |  | 0,379 |  | -0,058 |  | 0,696 |
| **Frontal_Inf_Orb_R** | -0,170 |  | 0,370 |  | 0,086 |  | 0,642 |  | -0,249 |  | 0,196 |  | -0,235 |  | 0,218 |  | -0,309 |  | 0,112 |  | -0,055 |  | 0,738 |
| **Frontal_Sup_Medial_L** | -0,311 |  | 0,100 |  | 0,006 |  | 0,984 |  | -0,178 |  | 0,339 |  | -0,179 |  | 0,335 |  | -0,207 |  | 0,264 |  | -0,034 |  | 0,810 |
| **Frontal_Sup_Medial_R** | -0,206 |  | 0,254 |  | 0,259 |  | 0,163 |  | 0,061 |  | 0,760 |  | -0,054 |  | 0,759 |  | 0,000 |  | 1,000 |  | 0,284 |  | 0,100 |
| **Frontal_Mid_Orb_L** | -0,002 |  | 0,995 |  | 0,215 |  | 0,254 |  | 0,103 |  | 0,612 |  | 0,049 |  | 0,781 |  | 0,205 |  | 0,270 |  | 0,023 |  | 0,958 |
| **Frontal_Mid_Orb_R** | -0,103 |  | 0,594 |  | 0,250 |  | 0,192 |  | 0,142 |  | 0,467 |  | 0,044 |  | 0,850 |  | 0,201 |  | 0,292 |  | 0,158 |  | 0,406 |
| **Gyrus_Rectus_L** | -0,122 |  | 0,516 |  | 0,225 |  | 0,230 |  | -0,084 |  | 0,627 |  | -0,154 |  | 0,415 |  | -0,068 |  | 0,701 |  | 0,067 |  | 0,774 |
| **Gyrus_Rectus_R** | -0,093 |  | 0,620 |  | 0,218 |  | 0,259 |  | -0,178 |  | 0,354 |  | -0,152 |  | 0,433 |  | -0,249 |  | 0,196 |  | -0,038 |  | 0,788 |
| **Insula_L** | -0,172 |  | 0,364 |  | -0,026 |  | 0,882 |  | -0,096 |  | 0,608 |  | -0,083 |  | 0,649 |  | -0,090 |  | 0,628 |  | -0,067 |  | 0,692 |
| **Insula_R** | -0,208 |  | 0,285 |  | 0,029 |  | 0,863 |  | 0,217 |  | 0,236 |  | 0,207 |  | 0,271 |  | 0,140 |  | 0,469 |  | 0,184 |  | 0,324 |
| **Cingulum_Ant_L** | -0,074 |  | 0,724 |  | 0,301 |  | 0,107 |  | 0,023 |  | 0,931 |  | -0,053 |  | 0,756 |  | 0,063 |  | 0,767 |  | 0,095 |  | 0,667 |
| **Cingulum_Ant_R** | -0,188 |  | 0,324 |  | 0,251 |  | 0,182 |  | 0,073 |  | 0,716 |  | 0,045 |  | 0,798 |  | 0,043 |  | 0,862 |  | 0,107 |  | 0,586 |
| **Cingulum_Mid_L** | -0,112 |  | 0,561 |  | 0,311 |  | 0,098 |  | 0,051 |  | 0,791 |  | -0,104 |  | 0,572 |  | 0,158 |  | 0,401 |  | 0,167 |  | 0,392 |
| **Hippocampus_L** | 0,310 |  | 0,097 |  | -0,018 |  | 0,919 |  | -0,048 |  | 0,798 |  | -0,179 |  | 0,354 |  | 0,107 |  | 0,570 |  | 0,036 |  | 0,855 |
| **Hippocampus_R** | 0,255 |  | 0,180 |  | 0,251 |  | 0,174 |  | 0,065 |  | 0,751 |  | -0,055 |  | 0,750 |  | 0,200 |  | 0,283 |  | 0,084 |  | 0,702 |
| **ParaHippocampal_L** | **0,385** |  | **0,035** |  | 0,115 |  | 0,546 |  | 0,274 |  | 0,136 |  | 0,193 |  | 0,304 |  | 0,286 |  | 0,120 |  | 0,232 |  | 0,214 |
| **ParaHippocampal_R** | **0,473** |  | **0,010** |  | 0,157 |  | 0,408 |  | 0,312 |  | 0,080 |  | 0,235 |  | 0,208 |  | 0,277 |  | 0,143 |  | 0,291 |  | 0,124 |
| **Amygdala_L** | **0,362** |  | **0,047** |  | 0,070 |  | 0,732 |  | 0,045 |  | 0,814 |  | -0,104 |  | 0,573 |  | 0,084 |  | 0,645 |  | 0,231 |  | 0,219 |
| **Amygdala_R** | 0,272 |  | 0,150 |  | 0,273 |  | 0,135 |  | 0,005 |  | 1,000 |  | -0,151 |  | 0,408 |  | 0,088 |  | 0,625 |  | 0,179 |  | 0,344 |
| **Caudate_L** | 0,215 |  | 0,261 |  | 0,061 |  | 0,762 |  | -0,097 |  | 0,616 |  | -0,192 |  | 0,305 |  | -0,077 |  | 0,693 |  | 0,101 |  | 0,603 |
| **Caudate_R** | 0,077 |  | 0,674 |  | 0,117 |  | 0,560 |  | -0,248 |  | 0,191 |  | -0,291 |  | 0,129 |  | -0,168 |  | 0,370 |  | -0,108 |  | 0,572 |
| **Putamen_L** | 0,017 |  | 0,930 |  | -0,113 |  | 0,563 |  | -0,091 |  | 0,623 |  | -0,066 |  | 0,728 |  | -0,096 |  | 0,609 |  | -0,074 |  | 0,679 |
| **Putamen_R** | 0,045 |  | 0,818 |  | -0,016 |  | 0,924 |  | -0,067 |  | 0,717 |  | -0,041 |  | 0,802 |  | -0,108 |  | 0,564 |  | -0,026 |  | 0,838 |
| **Temporal_Pole_Sup_L** | 0,146 |  | 0,423 |  | 0,038 |  | 0,843 |  | 0,149 |  | 0,438 |  | 0,181 |  | 0,343 |  | 0,107 |  | 0,578 |  | 0,049 |  | 0,859 |
| **Temporal_Pole_Sup_R** | 0,101 |  | 0,604 |  | 0,041 |  | 0,797 |  | 0,106 |  | 0,590 |  | 0,131 |  | 0,504 |  | 0,042 |  | 0,828 |  | 0,067 |  | 0,738 |
| **Temporal_Pole_Mid_L** | 0,128 |  | 0,509 |  | 0,187 |  | 0,326 |  | 0,142 |  | 0,453 |  | 0,000 |  | 1,000 |  | 0,197 |  | 0,284 |  | 0,237 |  | 0,203 |
| **Temporal_Pole_Mid_R** | -0,102 |  | 0,581 |  | 0,152 |  | 0,431 |  | -0,097 |  | 0,607 |  | -0,222 |  | 0,228 |  | -0,003 |  | 0,987 |  | 0,075 |  | 0,700 |

**Supplementary Table 2.** Structural connectivity correlation analysis for factors that may influence olfactory structural connectivity of the olfactory cortical network (OCN), including olfactory test scores (Sniffin’ Sticks TDI), subjective olfactory function (high vs. low group), and olfactory significance group (high vs. low group). Post hoc permutation tests (based on Pearson’s linear correlation coefficient) were performed to address the problem of multiple comparisons. While olfactory test scores and subjective assessment of olfactory function was not correlated with the strength of structural connectivity, the total olfactory significance score was associated with increased connectivity to parahippocampus bilaterally and left amygdala. TDI: combined olfactory threshold, discrimination, and identification score; _L: left; _R: right. For brain region abbreviations, see supplementary Table 3.

| **Region - Full** | **Region - Abbr. long** | **Region - Abbr. short** | **AAL label** |
| --- | --- | --- | --- |
| Superior frontal gyrus, dorsolateral part | Frontal_Sup_Orb | ORBsup | 5+6 |
| Superior frontal gyrus, orbital part | Frontal_Inf_Orb | ORBinf | 15+16 |
| Olfactory cortex | Olfactory | OC | 21+22* |
| Superior frontal gyrus, medial | Frontal_Sup_Med | SFGmed | 23+24 |
| Superior frontal gyrus, medial orbital | Frontal_Mid_Orb | ORBsupmed | 25+26 |
| Gyrus rectus | Rectus | REC | 27+28 |
| Insula | Insula | INS | 29+30 |
| Anterior cingulate and paracingulate gyri | Cingulum_Ant | ACG | 31+32 |
| Median cingulate and paracingulate gyri | Cingulum_Mid | DCG | 33+34 |
| Hippocampus | Hippocampus | HIP | 37+38 |
| Parahippocampal gyrus | ParaHippocampus | PHG | 39+40 |
| Amygdala | Amygdala | AMYG | 41+42 |
| Caudate nucleus | Caudate | CAU | 71+72 |
| Lenticular nucleus, putamen | Putamen | PUT | 73+74 |
| Thalamus | Thalamus | THA | 77+78 |
| Temporal pole: superior temporal gyrus | Temporal_Pole_Sup | TPOsup | 83+84 |
| Temporal pole: middle temporal gyrus | Temporal_Pole_Mid | TPOmid | 87+88 |

**Supplementary Table 3.** Brain regions and abbreviations. *The olfactory region used is an updated parcellation from Fjaeldstad et al. ^9^.Abbr: Abbreviation; AAL: Automated Anatomical Labeling.
